# The Partner Does Matter: The Structure of Heteroaggregates of Acridine Orange in Water

**DOI:** 10.3390/molecules24152816

**Published:** 2019-08-02

**Authors:** Ilya G. Shenderovich

**Affiliations:** Institute of Organic Chemistry, University of Regensburg, Universitaetstrasse 31, 93053 Regensburg, Germany; Ilya.Shenderovich@ur.de

**Keywords:** NMR spectroscopy, self-assembly, nitrogen heterocycles, fluorescent probes

## Abstract

Self-assembly of organic molecules in aqueous solutions is governed by a delicate entropy/enthalpy balance. Even small changes in their intermolecular interactions can cause critical changes in the structure of the aggregates and their spectral properties. The experimental results reported here demonstrate that protonated cations of acridine orange, acridine, and acridin-9-amine form stable J-heteroaggregates when in water. The structures of these aggregates are justified by the homonuclear ^1^H cross-relaxation nuclear magnetic resonance (NMR). The absorption and fluorescence of these aggregates deviate characteristically from the known H-homoaggregates of the protonated cations of acridine orange. The latter makes acridine orange a handy optical sensor for soft matter studies.

## 1. Introduction

The hydrophobic effect is the basis of self-assembly of organic molecules in and from aqueous solutions [1]. This process is governed by a delicate entropy/enthalpy balance and can be controlled using tuneable ion–ion interactions [2,3,4]. In turn, these interactions depend on tuneable molecular conformations [5,6]. In the case of dyes, such changes can be analyzed using optical spectroscopy [7,8,9]. The most obvious spectral signature of an aggregation is a shift of absorption contrary to the monomer absorption. The direction of the shift depends on the sign of the resonant electronic coupling in the given aggregate [10,11]. When the coupling is positive, the absorption maximum shifts to higher energies. Such ordering is called H-aggregates and corresponds to a roughly vertical stack of molecules. When the stack is in a tilted position—J-aggregates—the coupling is negative and the shift is reversed. Both types of aggregates can coexist in a solution [12] providing the opportunity to explore the entropy/enthalpy balance in depth [13]. The type of aggregation can be critically important for the efficiency of a given organic dye as a fluorescence sensor [14,15,16,17]. The position of the fluorescence maximum changes characteristically upon aggregation [18]. However, the intensity of the fluorescence is expected to be strongly reduced in H-aggregates [10,19]. The latter might mislead the interpretation of fluorescence experiments in complex systems.

**Figure 1:**
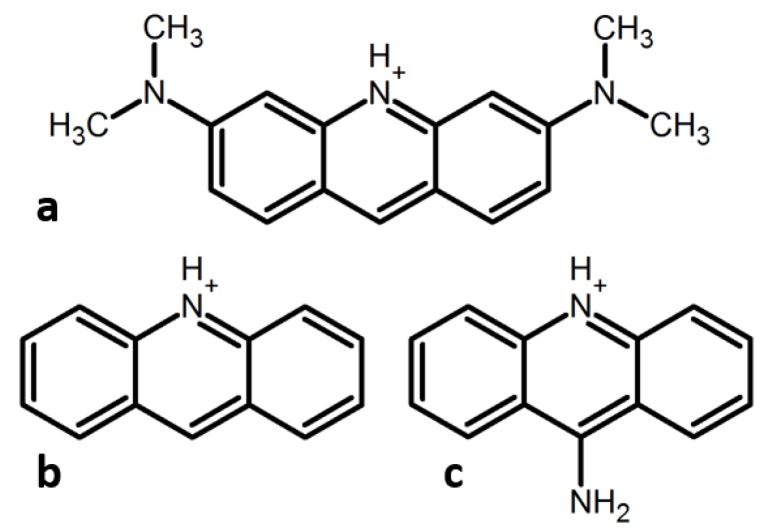
Protonated cations of acridines studied in this work. (**a**) *N,N,N′,N′*-tetramethylacridine-3,6-diamine (AOH^+^, AO = acridine orange), (**b**) acridine (AcrH^+^), (**c**) acridin-9-amine (9AAH^+^).

*N,N,N′,N′*-tetramethylacridine-3,6-diamine (acridine orange, AO) is one of the most studied organic dyes. The pKa value of AOH^+^ is 10.4 [20]. Thus, most studies research its cationic form AOH^+^ (Figure 1a). The dimerization constant of AOH^+^ in nonaqueous solutions is low [20,21]. In contrast, the oligomeric/monomeric ratio changes strongly in aqueous solutions in the concentration range 10^−5^–10^−4^ M [22]. Only H-aggregates have been observed up to date for AOH^+^ in water [23,24] and interfaces [25,26]. However, in all these cases, only the homoaggregation of AOH^+^ has been possible [27]. In complex molecular systems and in tissues, AOH^+^ may aggregate with other aromatic species. Neither the type nor spectral features of such aggregates can be predicted in advance. This paper reports the results of the experimental study of the heteroaggregation of AOH^+^ with protonated cations of acridine (AcrH^+^) and acridin-9-amine (9AAH^+^) (Figure 1b,c). The aggregate type is first identified using UV-VIS absorption and then justified by nuclear magnetic resonance (NMR). Fluorescence properties of these aggregates have also been characterized. For convenience sake, the most characteristic spectral signatures are collected at the end of the results section, Table 1.

## 2. Results

**Figure 2:**
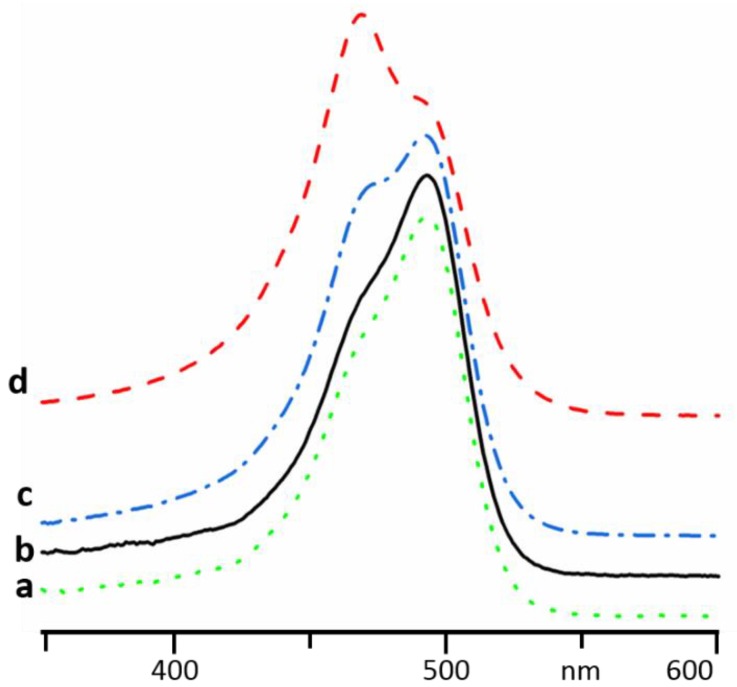
Evolution of the absorption of AOHCl in Milli-Q water upon an increase of concentration: 1 × 10^−6^ M (**a**), 1 × 10^−5^ M (**b**), 4 × 10^−5^ M (**c**), 1 × 10^−4^ M (**d**).

Absorption spectra of AOHCl in Milli-Q water at different concentrations are shown in Figure 2. Below 10^−5^ M, the maximum is located at 490 nm (Figure 2a,b). This maximum is attributed to AOH^+^ monomers. As the concentration is increased, this band decreases in intensity, giving rise to a band at 470 nm (Figure 2c,d). The new band is associated with aggregation of AOH^+^ into oligomers (presumably dimers) [19,22,28].

**Figure 3:**
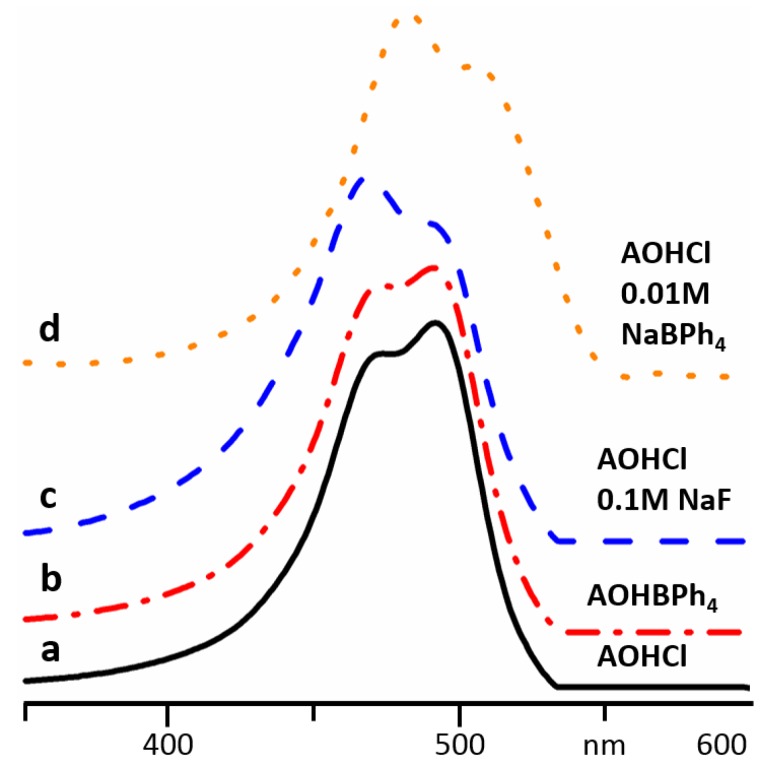
Absorption spectra of AOH^+^ in different aqueous solutions: 1 × 10^−5^ M of AOHCl (**a**), 1 × 10^−5^ M of AOHB(C_6_H_5_)_4_ (**b**), 1 × 10^−5^ M of AOHCl and 0.1 M of NaF (**c**), 1 × 10^−5^ M of AOHCl and 0.01 M of NaB(C_6_H_5_)_4_ (**d**).

At low concentrations, the absorption of (AOH)^+^R^-^ does not depend on the nature of R^-^. For example, the absorption spectra of AOHCl and AOHB(C_6_H_5_)_4_ at 10^−5^ M were equal (Figure 3a,b). At such concentrations, the salts were completely dissociated and the ions were solvated and did not interact specifically. The situation changes when specific ion effects cannot be ignored. In a 0.1 M aqueous solution of NaF, AOH^+^ was already aggregated at 10^−5^ M (Figure 3c). The same behavior was observed for other small ions [19]. In contrast, in concentrated solutions of inorganic salts of large and weakly coordinating anions, for example, I^−^, (BF_4_)^−^ and (ClO_4_)^−^, AOH^+^ formed contact pairs, giving rise to a broad absorption shifted to shorter wavelengths. Such complexes were studied in detail for a number of organic dyes [19,29] and are not the subject of this study.

This work studies the interaction of AOH^+^ with organic molecules. In a 0.01 M aqueous solution of NaB(C_6_H_5_)_4_ and 10^−5^ M of AOH^+^, the absorption spectrum resembles the one in 0.1 M of NaF but is shifted by 10 nm to longer wavelengths (Figure 3d).

**Figure 4:**
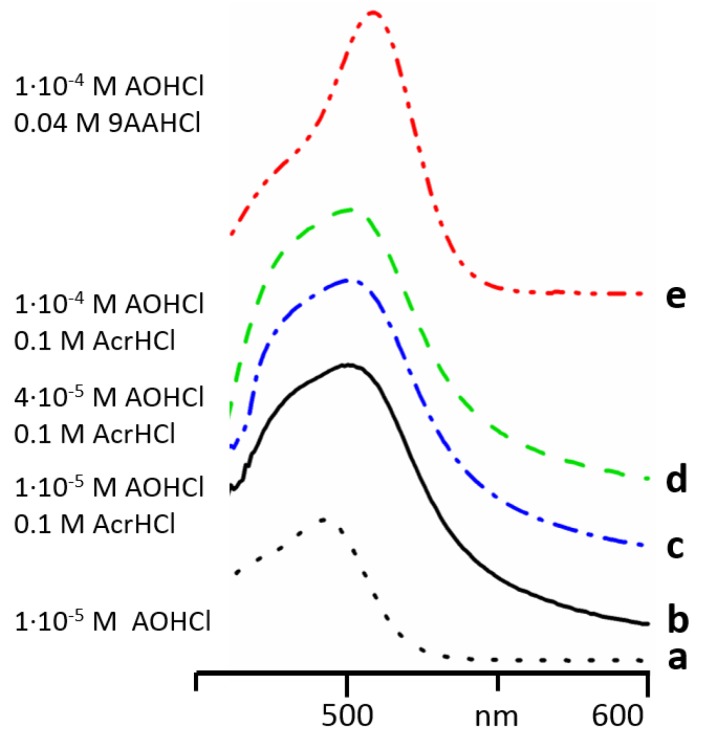
Absorption spectra of AOH^+^ in different aqueous solutions: 1 × 10^−5^ M of AOHCl (**a**), 1 × 10^−5^ M of AOHCl and 0.1 M of AcrHCl (**b**), 4 × 10^−5^ M of AOHCl and 0.1 M of AcrHCl (**c**), 1 × 10^−4^ M of AOHCl and 0.1 M of AcrHCl (**d**), 1 × 10^−4^ M of AOHCl and 0.04 M of 9AAHCl (**e**).

In Figure 4, absorption spectra of AOH^+^ measured in concentrated aqueous solutions of AcrHCl and 9AAHCl are shown. At these concentrations, AcrH^+^ and 9AAH^+^ absorbed strongly below 467 nm and 459 nm, respectively. Therefore, no reliable measurements are possible at short wavelengths, (Figure S1). However, it is more important to keep the total concentration of acridines similar in optical and NMR samples. Thus, the reported results are qualitative but not quantitative.

At these conditions, the spectra of AOH^+^ remarkably deviate from the absorption of AOH^+^ monomers (Figure 4a). Thus, AOH^+^ forms with AcrH^+^ and 9AAH^+^ heteroaggregates. The structure of the AOH^+^/AcrH^+^ heteroaggregates does not depend on AOH^+^ concentration (Figure 4b–d). The absorption maxima of AOH^+^ in the heteroaggregates with AcrH^+^ and 9AAH^+^ were 501 and 508 nm, respectively (Figure 4d,e).

**Figure 5:**
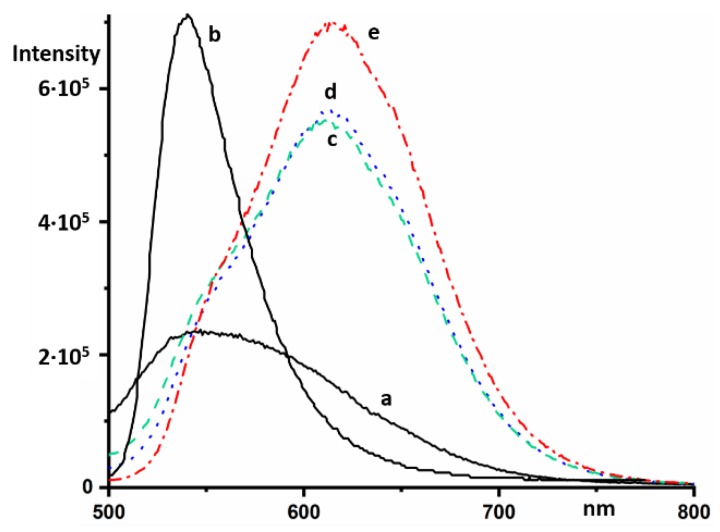
Steady-state fluorescence emission spectra in different aqueous solutions of NaB(C_6_H_5_)_4_ and AOHCl at an excitation wavelength of 470 nm: 0.02 M of NaB(C_6_H_5_)_4_ (**a**), 2 × 10^−5^ M of AOHCl (**b**), 2 × 10^−5^ M of AOHCl and 0.02 M of NaB(C_6_H_5_)_4_ (**c**), 2 × 10^−5^ M of AOHCl and 0.013 M of NaB(C_6_H_5_)_4_ (**d**), 4 × 10^−5^ M of AOHCl and 0.013 M of NaB(C_6_H_5_)_4_ (**e**).

Monomers and oligomers of AOH^+^ fluoresce in an aqueous solution at 529 and 634 nm, respectively [18]. The intensity of the latter is strongly reduced [19]. In a concentrated solution of NaB(C_6_H_5_)_4_, AOH^+^ fluoresces at 615 nm (Figure 5). The intensity of this fluorescence at an excitation wavelength of 470 nm was much stronger than the fluorescence of NaB(C_6_H_5_)_4_ (Figure 5a), and can be identified by changing the relative concentration of the components (Figure 5c–e).

**Figure 6:**
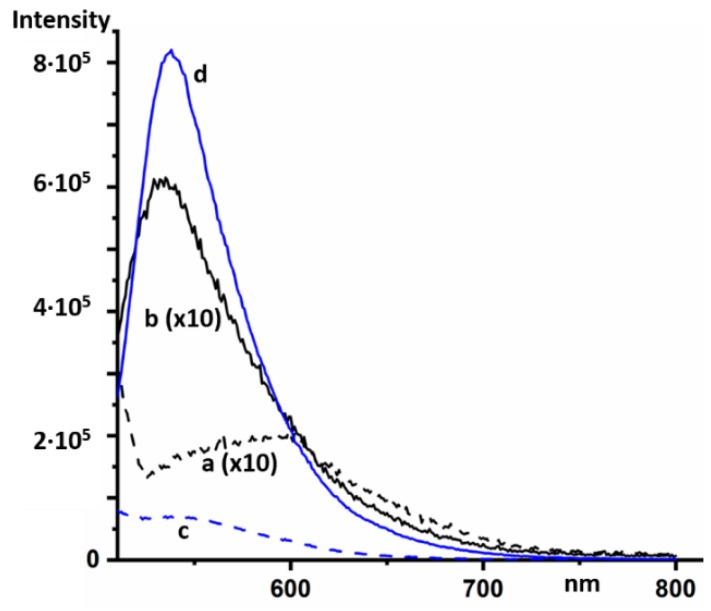
Steady-state fluorescence emission spectra in concentrated aqueous solutions of AcrHCl (0.1 M) at an excitation wavelength of 500 nm in the absence (**a**) and presence of 2 × 10^−5^ M of AOHCl (**b**). The intensity of the spectra was increased by a factor of ten. Steady-state fluorescence spectra in concentrated aqueous solutions of 9AAHCl (0.04 M) at an excitation wavelength of 500 nm in the absence (**c**) and presence of 2 × 10^−5^ M of AOHCl (**d**).

At low concentrations, the fluorescence maximum of AcrH^+^ in aqueous solutions appears at 475 nm [30,31]. A similar spectrum is observed in organic solvents [32]. AcrH^+^ absorbs below 485 nm [30,33]. However, at high concentrations, AcrH^+^ fluoresced in an aqueous solution at an excitation wavelength of 500 nm at 600 nm (Figure 6a). A stronger fluorescence of AOH^+^ in the same solution was located at 534 nm (Figure 6b).

At low concentrations, the emission spectrum of 9AAH^+^ exhibits a shoulder up to 600 nm [34,35]. At high concentrations, this shoulder was also present at an excitation wavelength of 500 nm (Figure 6c). A very strong fluorescence of AOH^+^ in the same solution was located at 538 nm (Figure 6d).

**Figure 7:**
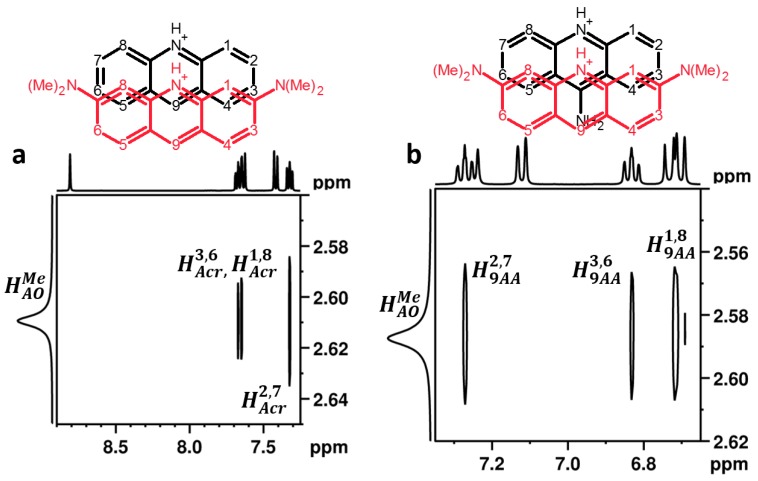
2D rotating frame nuclear Overhauser effect spectroscopy (ROESY) ^1^H nuclear magnetic resonance (NMR) spectra of AOHCl/AcrHCl (**a**) and AOHCl/9AAHCl (**b**) mixtures (molar ratio 1:1) in D_2_O. The numbering of protons is the same as that of the attached carbon atoms.

Figure 7 shows a selected part of 2D rotating frame nuclear Overhauser effect spectroscopy (ROESY) ^1^H NMR spectra of AOHCl/AcrHCl and AOHCl/9AAHCl mixtures at a molar ratio of 1:1 in D_2_O. This method establishes correlations between nuclei, which are close in space. Besides intramolecular correlations (not shown), there are a number of intermolecular correlations. For the AOHCl/AcrHCl mixture, the strongest among them were between AOH^+^ methyl groups and AcrH^+^ protons 1 and 8, 2 and 7, and 3 and 6. The most expected structure of this heteroaggregate is shown in Figure 7a. For the AOHCl/9AAHCl mixture, the strongest intermolecular correlations were observed between AOH^+^ methyl groups and 9AAH^+^ protons 1 and 8, 2 and 7, and 3 and 6. The most expected structure of this heteroaggregate is shown in Figure 7b.

## 3. Discussion

Both UV-VIS absorption and NMR correlation denoted that heteroaggregates of AOH^+^ with AcrH^+^, as well as with 9AAH^+^, were of the J-type. The absorption maxima of AOH^+^ in these J-heteroaggregates were shifted to longer wavelengths by 10–20 nm compared to AOH^+^ monomers, and by 40 nm compared to AOH^+^ H-homoaggregates. The J-heteroaggregates under question fluoresced intensively at similar wavelengths as AOH^+^ monomers, that is, their fluorescence was shifted to shorter wavelengths by 100 nm compared to AOH^+^ H-homoaggregates. Thus, the type of aggregation can be controlled by optical methods.

The question arises as to why the structure of the aggregates critically depends on small changes in the structure of the partner. The electrostatic potential map was similar in all three acridines (Figure S2). Therefore, the electrostatic interactions in all these aggregates should be similar as well. In contrast, steric hindrance between methyl groups of two neighboring AOH^+^ might be important. Indeed, it has been shown in the past for different dyes of the AO-type core structure (three fused aromatic rings, a central heteroatom, and 2,7-NR2 substituents) that the angles between the dye transition moments in their homoaggregates are 30°–60°, that is, the molecules in the aggregates are on top of each other but are rotated [23]. The new data suggest that in the heteroaggregates of AOH^+^ with protonated 2,7-nonsubstituted acridines, the molecules are shifted along the symmetry axis but not necessarily rotated (Figure 7). Of course, these expected structures should not be understood as rigid ones. The reported optical data denote that when the concentration of protonated acridines in water is above a certain critical value all these cations are aggregated. Molecular exchange between these aggregates and intra-aggregate dynamics are yet to be studied. The reported NOE data suggest only that the probability to find AOH^+^ methyl groups close to AcrH^+^ and 9AAH^+^ protons 1, 8, 2, 7, 3, and 6 is higher than for 4, 5, and 9 protons. The structure of the H-homoaggregates of AOH^+^ cannot be studied using NMR without a specific isotope labeling of a part of the molecules. The most promising procedure is a partial ^13^C labeling of methyl groups.

The ROESY NMR technique was only successful in providing intermolecular contacts that composed the structure of aggregates when both partners were acridines. No intermolecular contacts were detected in test experiments for aqueous solutions of AOHCl with quinoline-HCl and *N,N*-dimethylpyridin-4-amine-HCl. Dipole-dipole NMR is quite sensitive to intermolecular interactions [36,37]. Therefore, only protonated acridines form in water relatively stable aggregates.

The optical properties of AOH^+^ in concentrated solutions of B(C_6_H_5_)_4_^–^ strongly deviated from solutions of small and large inorganic anions. The absorption pattern of the former mixtures can be explained in two ways.

First, they can reflect an aggregation between the *π*-systems of AOH^+^ and B(C_6_H_5_)_4_^–^. Alternatively, they can be caused by the desolvation of the mobile proton of AOH^+^ because of its coordination between the phenyl rings of B(C_6_H_5_)_4_^–^. The interaction of anions of the B(C_6_H_5_)_4_^–^ type with protonated cations was weaker than of the BF_4_^–^ cation, even in organic solvents [38,39]. This argumentation supports the first scenario. The fluorescence spectrum of AOH^+^ in the concentrated solution of B(C_6_H_5_)_4_^–^ deviated from the one of J-heteroaggregates and was similar to the fluorescence of H-homoaggregates. If AOH^+^ could form with B(C_6_H_5_)_4_^–^ H-type heteroaggregates, it would support the first scenario as well. However, such heteroaggregates are very unlikely. Moreover, the fluorescence of H-aggregates was low while the fluorescence of AOH^+^ at high concentration of B(C_6_H_5_)_4_^–^ was intense. We can speculate that when B(C_6_H_5_)_4_^–^ is at large excess it interacts with AOH^+^ and expels water solvated the N–H proton. This situation resembles, in certain respects, the situation at silica surface where water cannot solvate silanol groups [40]. However, in contrast to better coordinating anions (for example, BF_4_^-^), B(C_6_H_5_)_4_^–^ attracts the N-H proton weaker than water. Thus, the N–H distance becomes shorter and both absorption and fluorescence move to longer wavelengths. This effect can be potentially studied by using the ^15^N NMR chemical shift that specifically depends on the N–H distance [41,42,43]. However, this experiment requires ^15^N-labeled AO.

## 4. Materials and Methods

UV-VIS spectra were recorded on a Cary-100 spectrometer (Agilent Technologies) using standard 10 mm (AOH^+^ concn. < 10^−4^ M) and 2 mm (AOH^+^ concn. ≥ 10^−4^ M) quartz cuvettes. Fluorescence spectra were measured on a FluoroMax-4 spectrofluorometer (Horiba Scientific). Nuclear magnetic resonance (NMR) experiments were performed at 300 K on a Bruker AVIII-HD-400 spectrometer (Bruker BioSpin) equipped with a temperature stabilization system. NMR samples for the ROESY (rotating frame overhause effect spectroscopy) experiments contained about 0.05 M of AOHCl and either 0.05 M of AcrHCl or 0.05 M of 9AAHCl in 0.6 mL D_2_O. The mixing time was 200 ms, the relaxation time was 2 s, and the number of scans was 32. Chemicals were purchased from commercial suppliers and used without further purification. The pH of all used solutions was below 8. Optical properties of neutral and cationic forms of acridine derivatives differ characteristically [18,21,30,31,32]. Thus, there was no reason to control the pH of the solutions with a higher precision. The intensities of reported absorption spectra have been optimized for the best illustration of spectral changes. In order to stress this limitation, the ordinate axes are missing.

## 5. Conclusions

This work reports an experimental study of the aggregation of protonated acridine cations in aqueous solutions. The driving force of this hydrophobic assembly is neither a repulsion between water and heterocycles, nor an attraction of the heterocycles to each other. The reason is that the hydrogen bond network of water needs to be adopted to the solute [44]. It is the free energy of solvation that defines whether the aggregation happens or not. Another critically important role of water is solvation of the N–H proton. These water molecules are the integral part of the charged aggregates. This fact significantly complicates any modelling of the structure of such aggregates [45].

The obtained results demonstrate that the structure of the aggregates critically depends on small changes in molecular structure. It seems that when the aggregating species have bulky substituents at the positions 2 and 7, they form H-aggregates. In contrast, when one of the species does not have such substituents, J-aggregates are formed. Which of these aggregates is more thermodynamically stable at equal concentrations of the species needs yet to be studied. However, the stability of J-heteroaggregates formed by AOH^+^ with AcrH^+^ and 9AAH^+^ in aqueous solutions is high enough to study their structure by intermolecular dipole–dipole NMR.

The reported data show that the absorption and florescence properties of AOH^+^ depend in a characteristic way on both π–π aggregation and hydrogen bonding. The latter can be quantitatively characterized even for complex molecular systems in solutions [46,47], on surfaces [48,49], and in solids [50,51] using ^15^N-NMR. Thus, ^15^N labeled AO exhibits a very useful sensor to study the structure of soft matter.

## Figures and Tables

**Table 1:** Characteristic spectral signatures of AOH^+^ in different aqueous solutions.

Aggregate	Concentration, M		Absorption,nm	Fluorescence Emission, nm
	AOH^+^	Copartner		
Monomer	10^−5^	**−**	490	529 (λ_e x_= 492) [18]
Homoaggregates	10^−4^	−	470	634 (λ_ex_ = 470) [18]
Heteroaggregates with AcrH^+^	10^−5^	10^−1^	501	534 (λ_ex_ = 500)
Heteroaggregates with 9AAH^+^	10^−5^	4 × 10^−2^	508	538 (λ_ex_ = 500)
Aggregates with [B(C_6_H_5_)_4_]^-^	10^−5^	10^−2^	480 and 460	615 (λ_ex_ = 470)

## Supplementary Materials

The following are available online, Figure S1: Absorption spectra of 0.1 M of AcrHCl (dashed) and 0.04 M of 9AAHCl (dotted) in water, Figure S2: The electrostatic potential map of AOH^+^, AcrH^+^, and 9AAH^+^ calculated at the B3LYP/aug-cc-pVTZ approximation, Figure S3: ^1^H NMR spectra of AOHCl in D_2_O at 0.001 M (**a**) and 0.1 M (**b**), Figure S4: ^1^H and HSQC NMR spectra of an AOHCl/9AAHCl mixture in D_2_O, Figure S5: ^1^H and HMBC NMR spectra of an AOHCl/9AAHCl mixture in D_2_O, Figure S6: ^1^H NMR spectrum of an AOHCl/AcrHCl mixture in D_2_O, Figure S7: Normalized excitation spectrum of a mixture of 2·10^−5^ M of AOHCl and 0.02 M of NaB(C_6_H_5_)_4_, λ_ex_=614 nm, Figure S8: Normalized excitation spectrum of a concentrated aqueous solution of AcrHCl (0.1 M) and 2·10^−5^ M of AOHCl, λ_ex_=541 nm, Figure S9: Normalized excitation spectrum of a concentrated aqueous solution of 9AAHCl (0.04 M) and 2·10^−5^ M of AOHCl, λ_ex_ = 550 nm.
