# Supplementary material for: The Partner Does Matter: The Structure of Heteroaggregates of Acridine Orange in Water
Source: Molecules. 2019 Aug 2;24(15):2816. doi: 10.3390/molecules24152816 (PMC6696166; doi:10.3390/molecules24152816)
Supplement: Supplementary file 1 [file molecules-24-02816-s001.pdf]

Supporting Information for Publication

The partner does matter: the structure of heteroaggregates of acridine orange in water

Ilya G. Shenderovich

Institute of Organic Chemistry, University of Regensburg, Universitaetstrasse 31, 93053 Regensburg, Germany

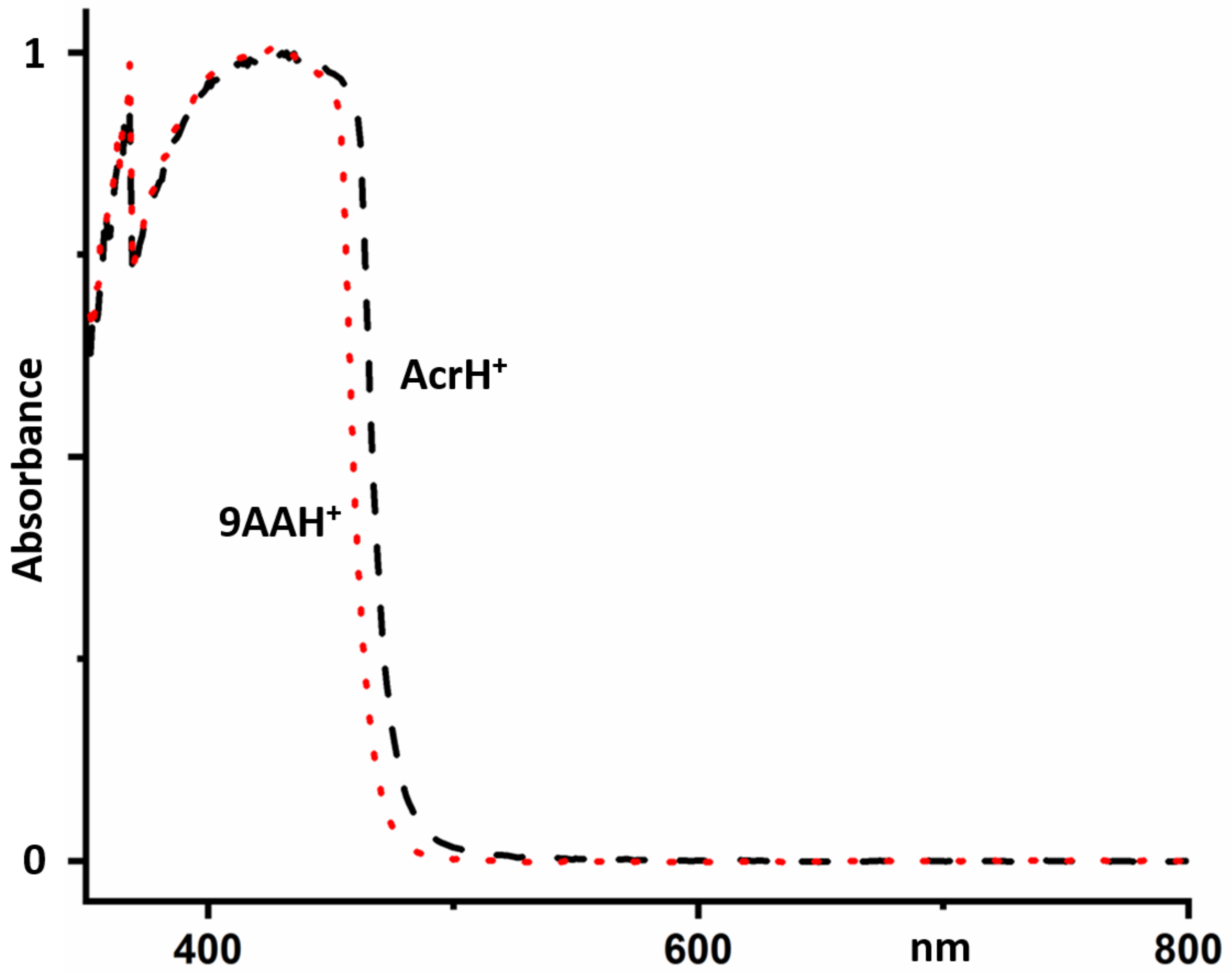

Figure S1. Absorption spectra of 0.1 M of AcrHCl (dashed) and 0.04 M of 9AAHCl (dotted) in water.

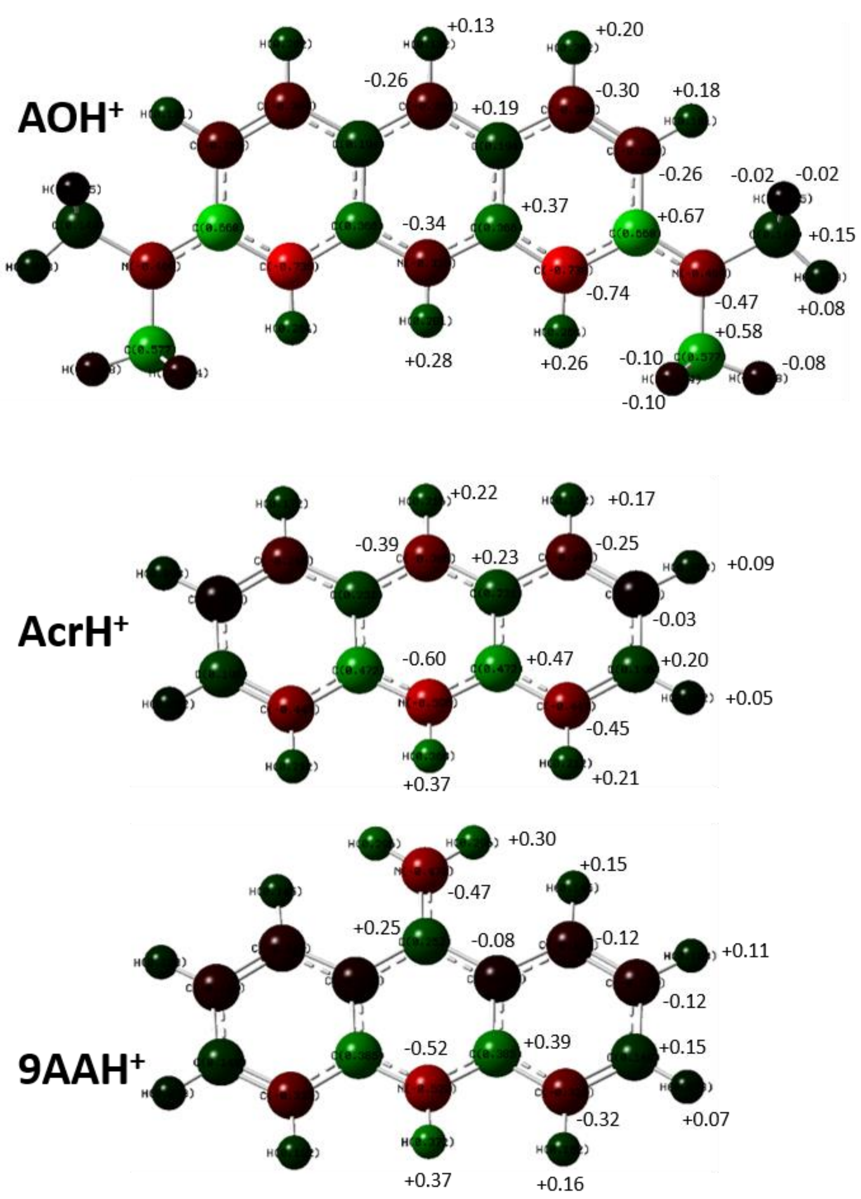

Figure S2. The electrostatic potential map of AOH^+, $AcrH^+$, and $9AAH^+$ calculated at the B3LYP/aug-cc-pVTZ approximation using Gaussian 16.A.03[1] according to L. E. Chirlian and M. M. Francl, "Atomic charges derived from electrostatic potentials – a detailed study," J. Comp. Chem., 8 (1987) 894-905. DOI: 10.1002/jcc.540080616. Charge densities are shown in numbers and colors: positive - bright green, negative - bright red, neutral– dark.

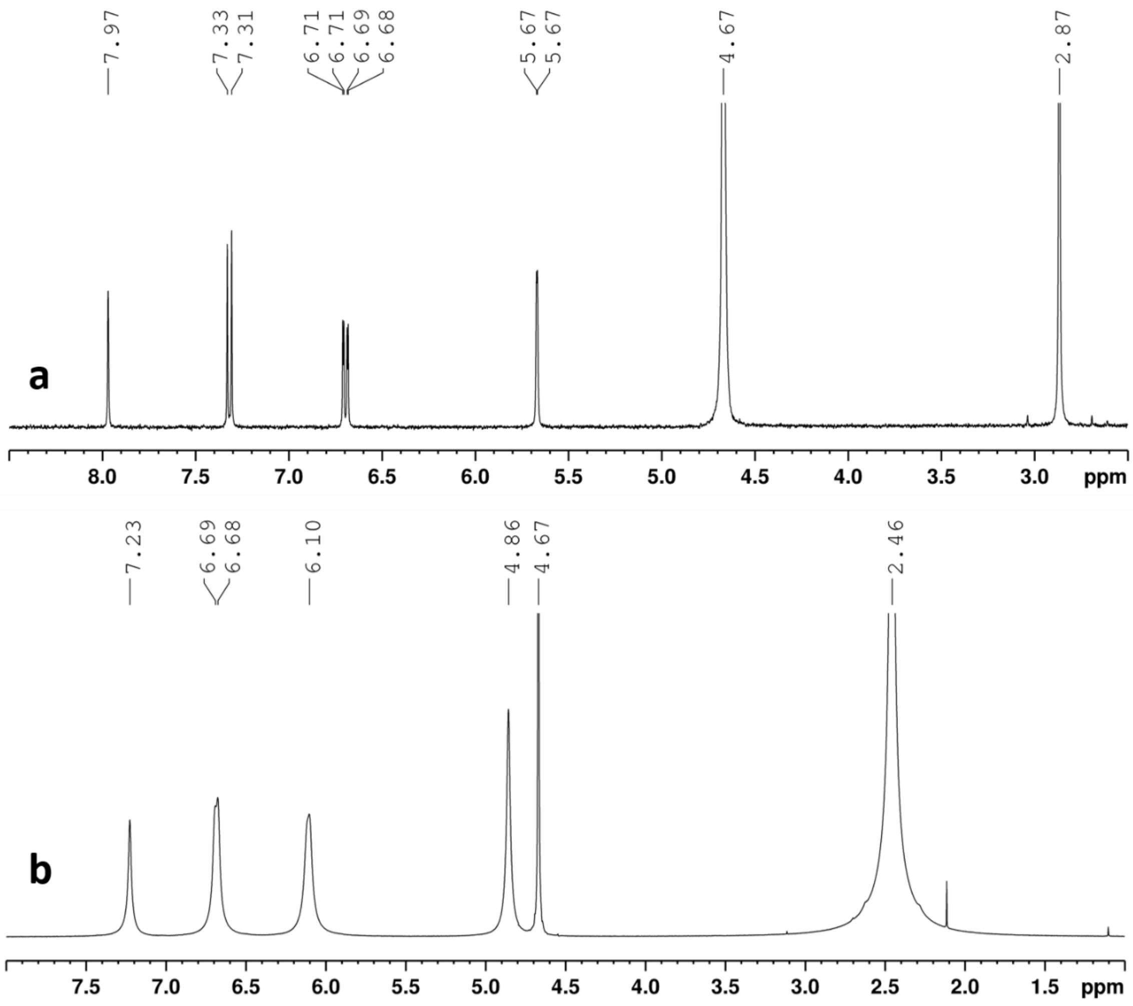

Figure S3. ^{1}H NMR spectra of AOHCl in D_2O at 0.001 M (a) and 0.1 M (b).

Figure S4. ^{1}H and HSQC NMR spectra of an AOHCl/9AAHCl mixture in D_2O.

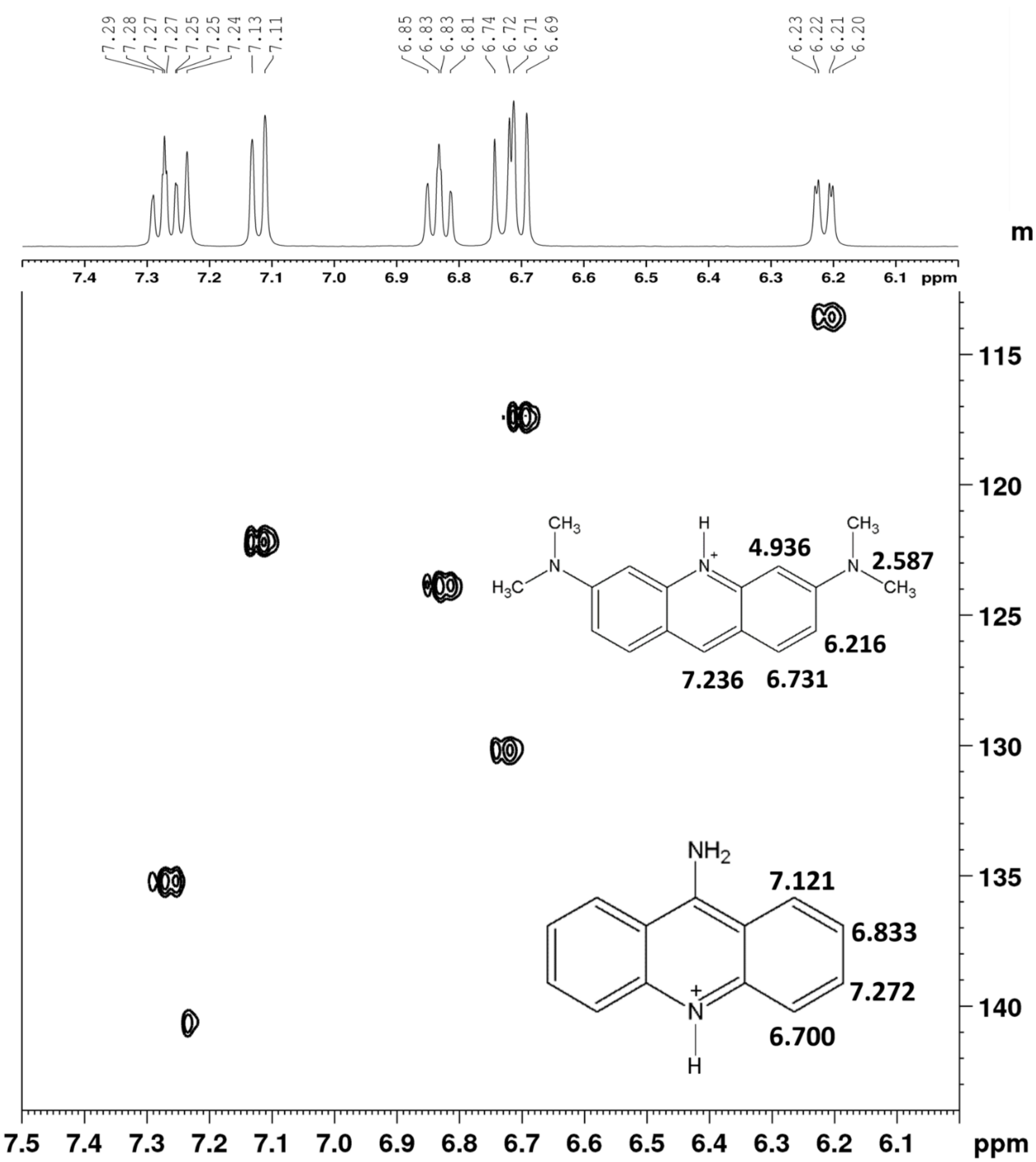

Figure S5. ^{1}H and HMBC NMR spectra of an AOHCl/9AAHCl mixture in D_2O.

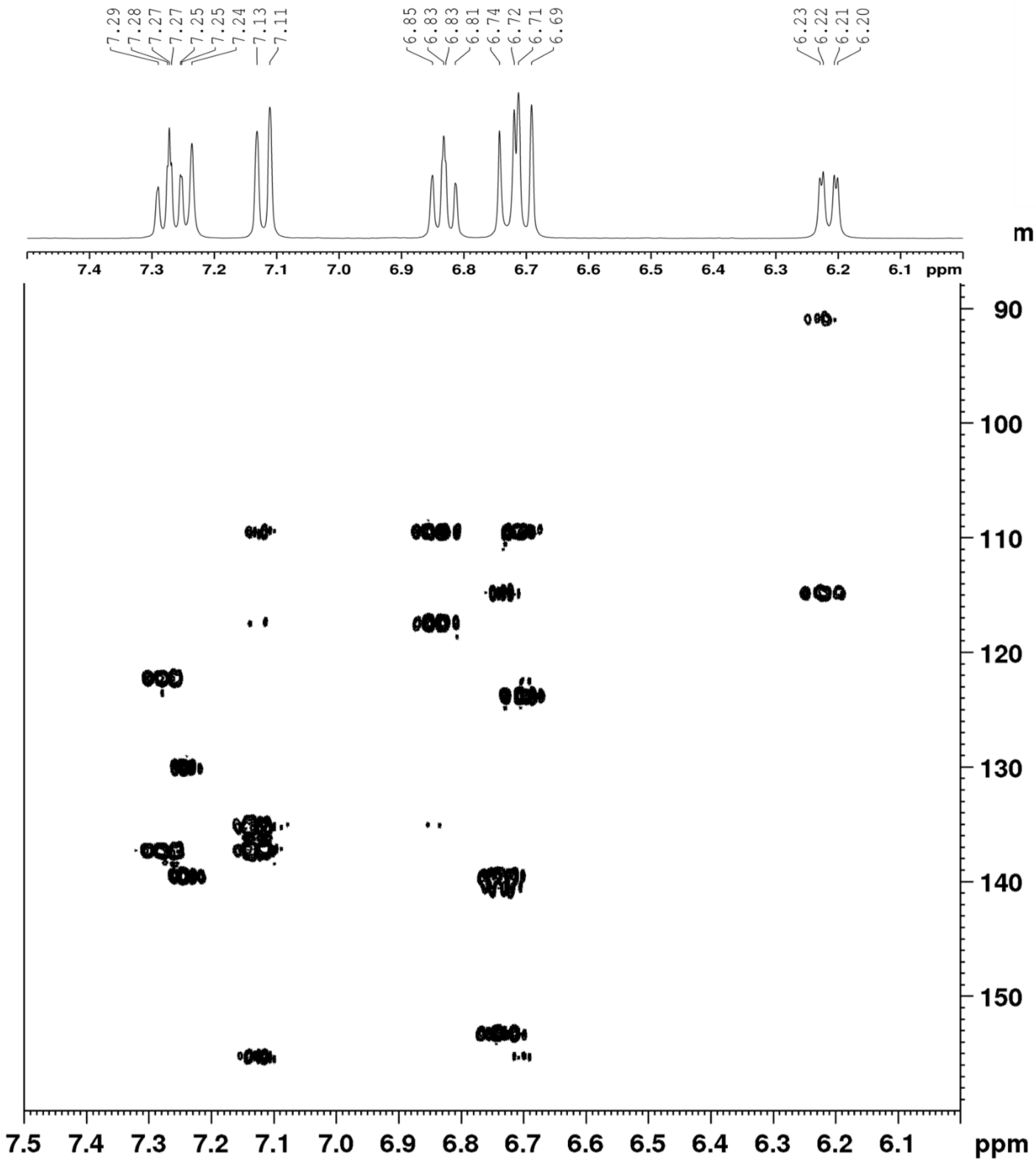

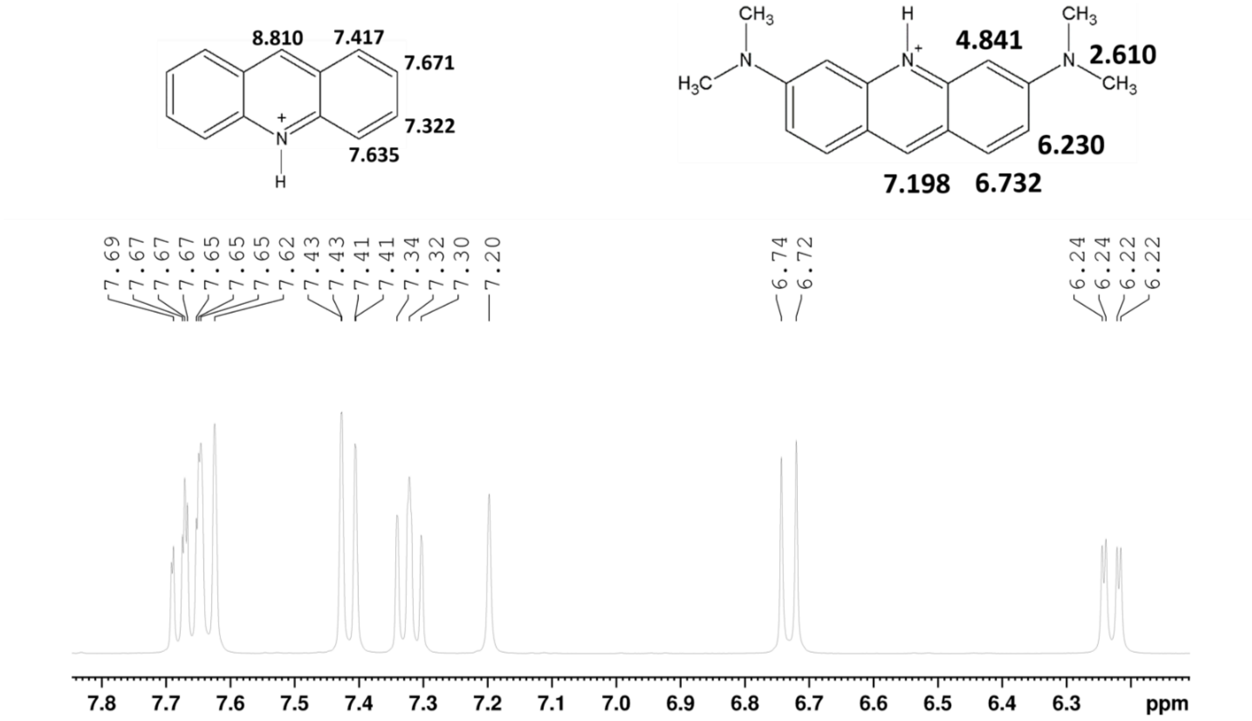

Figure S6. ^{1}H NMR spectrum of an AOHCl/AcrHCl mixture in D_2O.

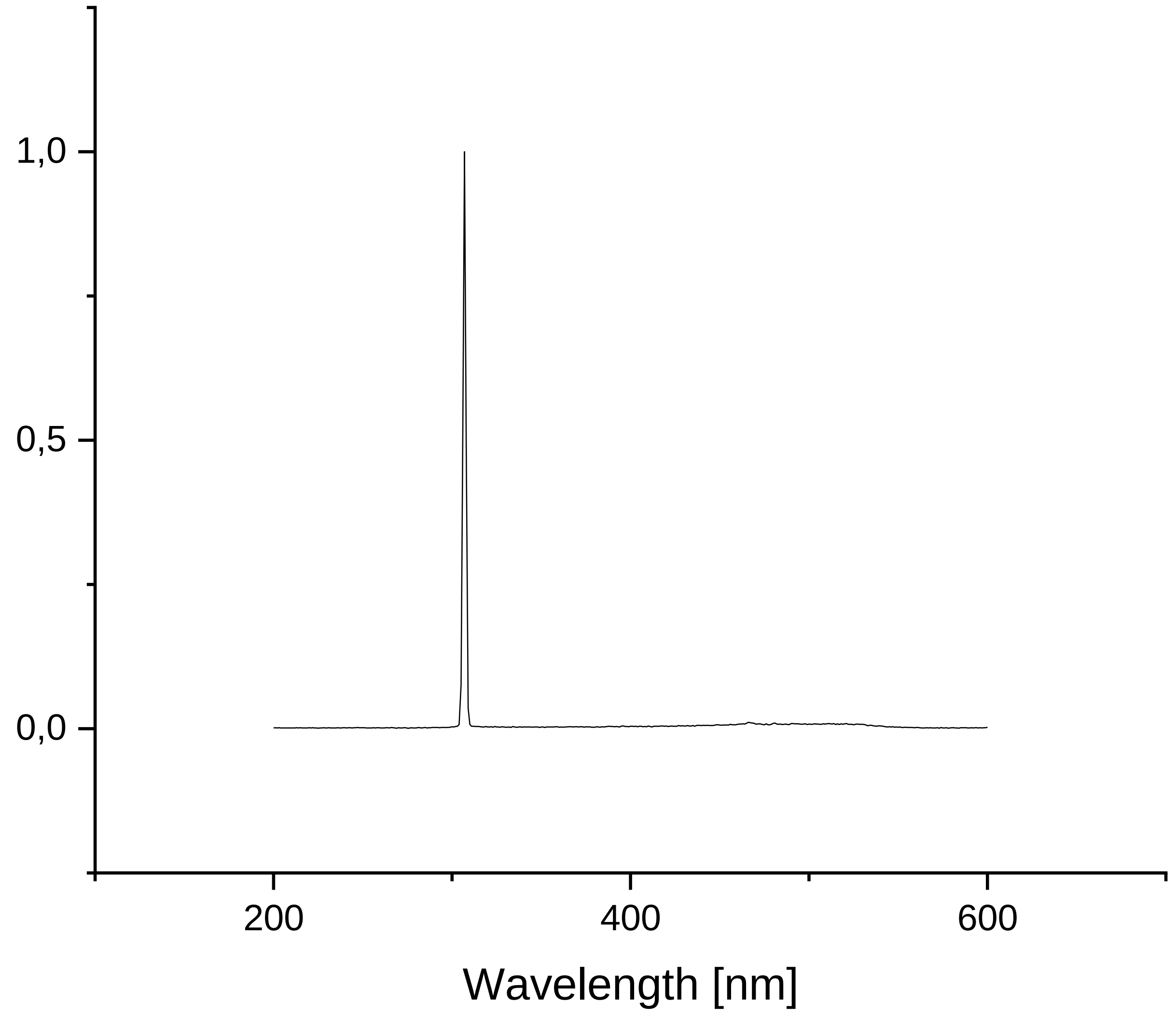

Figure S7. Normalized excitation spectrum of a mixture of $2 \cdot 10^{-5}$ M of AOHCl and 0.02 M of $NaB(C_6H_5)_4$, λ_{ex}=614 nm.

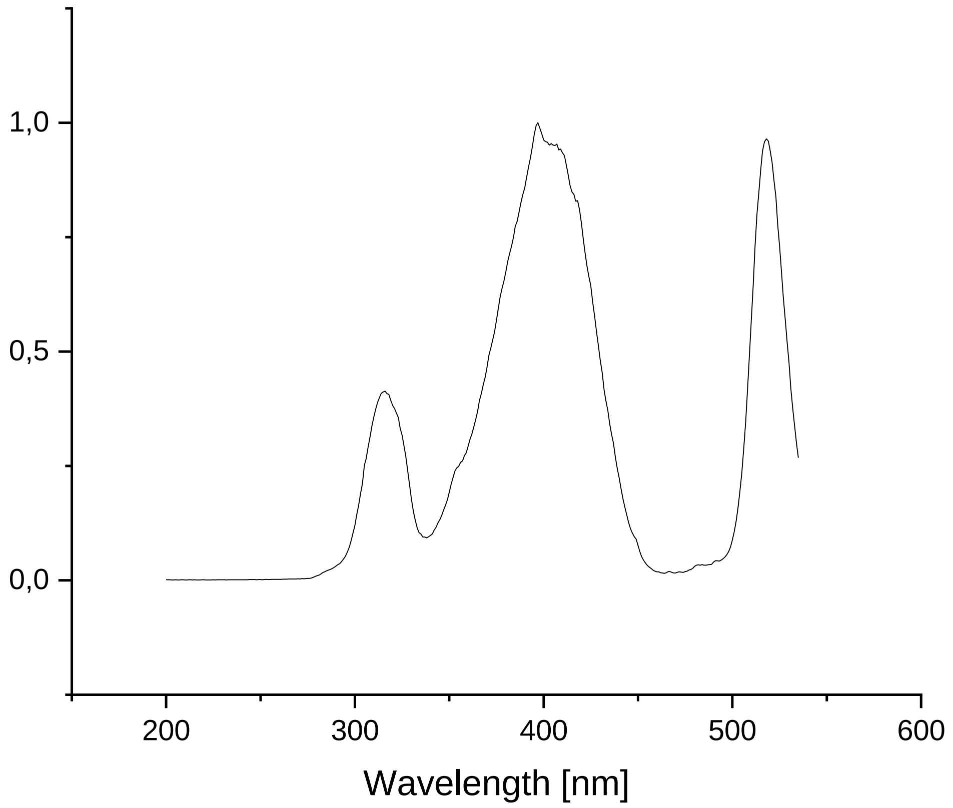

Figure S8. Normalized excitation spectrum of a concentrated aqueous solution of AcrHCl (0.1 M) and $2\cdot10^{-5}$ M of AOHCl, λ_{ex}=541 nm.

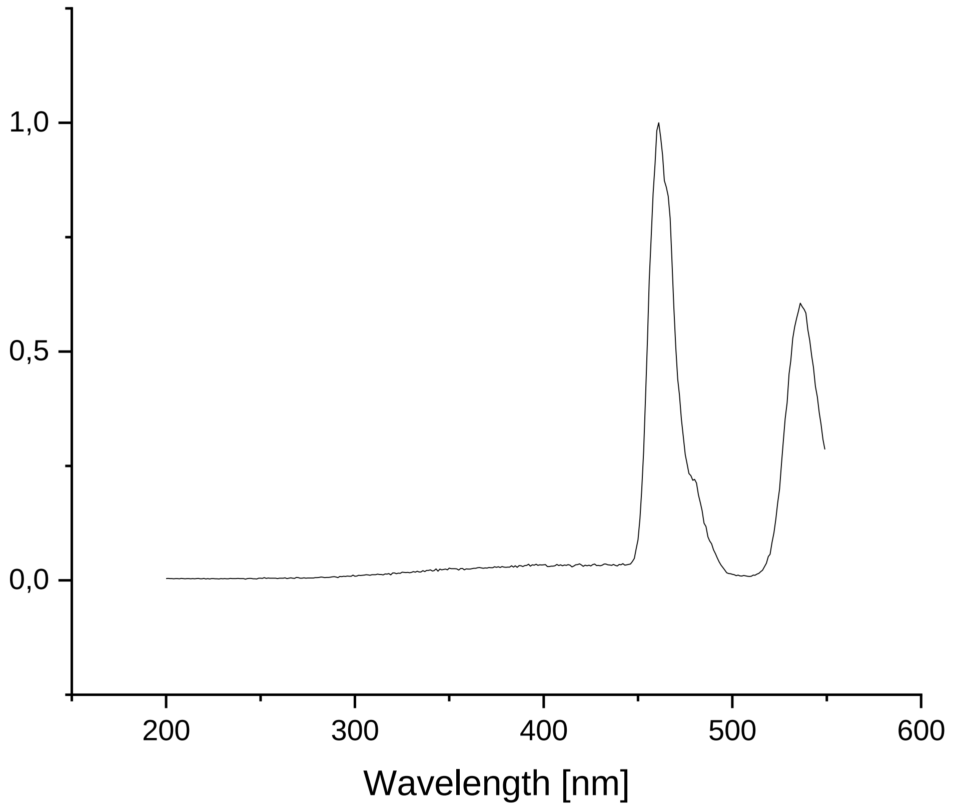

Figure S9. Normalized excitation spectrum of a concentrated aqueous solution of 9AAHCl (0.04 M) and $2 \cdot 10^{-5}$ M of AOHCl, λ_{ex}=550 nm.

---

[1] Gaussian 16, Revision A.03, M. J. Frisch, G. W. Trucks, H. B. Schlegel, G. E. Scuseria, M. A. Robb, J. R. Cheeseman, G. Scalmani, V. Barone, G. A. Petersson, H. Nakatsuji, X. Li, M. Caricato, A. V. Marenich, J. Bloino, B. G. Janesko, R. Gomperts, B. Mennucci, H. P. Hratchian, J. V. Ortiz, A. F. Izmaylov, J. L. Sonnenberg, D. Williams−Young, F. Ding, F. Lipparini, F. Egidi, J. Goings, B. Peng, A. Petrone, T. Henderson, D. Ranasinghe, V. G. Zakrzewski, J. Gao, N. Rega, G. Zheng, W. Liang, M. Hada, M. Ehara, K. Toyota, R. Fukuda, J. Hasegawa, M. Ishida, T. Nakajima, Y. Honda, O. Kitao, H. Nakai, T. Vreven, K. Throssell, J. A. Montgomery, Jr., J. E. Peralta, F. Ogliaro, M. J. Bearpark, J. J. Heyd, E. N. Brothers, K. N. Kudin, V. N. Staroverov, T. A. Keith, R. Kobayashi, J. Normand, K. Raghavachari, A. P. Rendell, J. C. Burant, S. S. Iyengar, J. Tomasi, M. Cossi, J. M. Millam, M. Klene, C. Adamo, R. Cammi, J. W. Ochterski, R. L. Martin, K. Morokuma, O. Farkas, J. B. Foresman, and D. J. Fox, Gaussian, Inc., Wallingford CT (2016).
